# Contextual and time dependent pain in fibromyalgia: An explorative study

**DOI:** 10.1186/1756-0500-5-644

**Published:** 2012-11-20

**Authors:** Egil A Fors, Tormod Landmark, Øyvind Bakke

**Affiliations:** 1 Department of Psychiatry, St Olav University Hospital, Trondheim, Norway; 2 Department of Public Health and General Practice, Faculty of Medicine, General Practice Research Unit, Norwegian University of Science and Technology, Trondheim, Norway; 3 National Competence Centre for Complex Symptom Disorders, St Olav University Hospital, Trondheim, Norway; 4 Department of Mathematical Sciences, |Norwegian University of Science and Technology, Trondheim, Norway

**Keywords:** Fibromyalgia, Chronic pain, Context, Time, Long-term, Cohort, Explorative

## Abstract

**Background:**

Little is known about contextual effects on chronic pain, and how vulnerability factors influence pain in different contexts. We wanted to examine if fibromyalgia (FM) pain varied between two social contexts, i.e. at home versus in a doctor office, when it was measured the same day, and if pain was stable for 14 years when measured in similar contexts (doctor office). Our secondary aim was to explore if pain vulnerability factors varied in the two different contexts.

**Findings:**

Fifty-five female FM patients were included in the study and scored pain in both contexts at baseline. Their age ranged between 21–68 years (mean 45.7), mean education level was 11 years and mean FM-duration was 15.6 years. Their mean pain was perceived significantly lower at home than in a doctor context the same day. However, pain was much more stable when measured in two similar contexts 14 year apart where 30 subjects (54.5%) completed. Predictor analyses revealed that pain vulnerability factors apparently varied by home and doctor contexts.

**Conclusion:**

Pain and pain predictors seem to vary by contexts and time, with less pain at home than to a doctor the same day, but with unchanged pain in the same context after 14 years. Thus, contextual pain cues should be accounted for when pain is measured and treated, e.g. by focusing more on home-measured pain and by optimizing the doctor office context. This explorative study should be followed up by a larger full-scale study.

## Findings

Fibromyalgia (FM) is a disorder characterized by chronic, widespread pain (CWP) with increased sensitivity for mechanical pressure in at least 11 out of 18 defined tender points (TP) [1]. FM is associated with female gender, low income and education, fatigue, irritable bowel and bladder symptoms, headache, negative mood and sleeplessness [2,3], previous pain [4] and stress induced HPA axis reactivity [5]. The prognosis is uncertain, and there are few and inconclusive prospective studies. FM pain appear stable from two to ten years in some natural course studies [6-13], while others demonstrate aggravation [14], improvement [15] or mixed aggravation and improvement [16]. Long-term follow-ups after psychological interventions have shown small effects on pain, but improvement in quality of life (QoL) [17]. Guided imagery has improved FM pain in the short term [18], but not in the long-term [19]. Cognitive behavioral therapy (CBT) has improved worry, pain behavior, pain coping, depression and reduced healthcare-seeking behavior in prospective studies [20,21].

According to the neuromatrix pain theory, the perception of pain is dynamically influenced by emotions, motivations, memory and other sensory and homeostatic inputs [22-24]. Thus, pain is always experienced in a context and influenced by it [25]. Context affects a variety of health measures including blood-pressure, which appears lower at home than to a doctor [26,27]. Also memory and learning [28] as well as anxiety and fear-conditioning [29,30] are affected by context. The impact of contextual factors for the experience of pain has been demonstrated in brain imaging studies and laboratory experiments [31,32]. For example, animal studies have shown that one drug has different effects in dissimilar contexts [33,34].

In a Norwegian study, healthy males reported less laboratory pain in a context with female investigators, compared to a context with male investigators, while females reported the same pain regardless of the investigators’ gender [35]. Perhaps the most obvious example of context influencing pain is the discrepancy observed in clinical and experimental contexts [36]. However, there are few studies investigating variations in the reporting of pain by contextual factors among patients with chronic pain. In one study, variations in weather were related to daily variations in pain among FM patients [37]. Other studies have shown that the social contexts are important for the acceptance of chronic pain [38] and the appraisals of pain threats [39].

In this study we monitored a cohort of female FM patients where pain was measured at home and in a doctor office the same day (baseline) and reexamined 14 years later. Our main research question was to examine if pain was context-dependent or time-dependent, i.e. if pain would vary by two different social contexts in one day and by a 14 years’ time lag from baseline to follow-up in one context. As a secondary aim we wanted to investigate if vulnerability factors were associated with pain in the different contexts and time points.

## Methods

### Design

This study is a prospective cohort with an explorative design, and the patients were followed from baseline in August 1991- May 1992 to follow-up in August 2005, and thus had no Clinicaltrials.gov number.

### Participants

**Figure 1:**
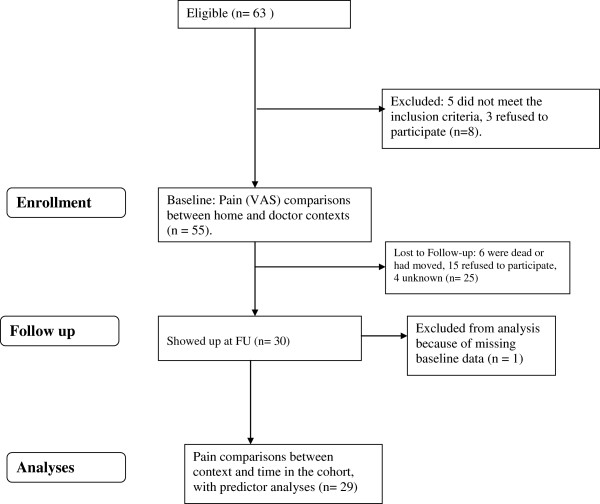
**A CONSORT-diagram of the study design.** FU = Follow-up.

55 female subjects were classified and found eligible for this study according to the American College of Rheumatology (ACR) criteria for FM, including chronic widespread pain (CWP) with at least 11 out of 18 specified tender points [1]. The women’s age at baseline ranged between 21–68 years (mean 45.7) and their mean education level was 11 years and their mean FM-duration was 15.6 years [26]. 14 years later, 30 of these 55 (54.5%) showed up to measure pain and other parameters after having been invited to a meeting after formal information; 15 refused to participate, 6 were dead or had moved while 4 were unknown. One was later excluded due to missing data (see Figure 1). Thus, n = 29 were included in the 14 years FU analyses.

### Procedure

A signed informed consent to participate was obtained before the patients scored their current pain twice in a 30 minutes interval (mean VAS) within a family doctor context (Pain DC) before the other baseline parameters were scored. Thereafter, they re-scored pain twice in their home context (Pain HC) one hour later. Following 30 days using amitriptyline/placebo medication and/or guided imagery/control programs, they measured post-score pain [18]. After these 30 days there were *no* active or passive interventions or attention from the experimentalists before the FU 14 years later. Then, the FM patients were contacted by mail and phone for FU examinations. They signed new informed consents before they re-scored pain (Pain FU) to the same doctor as at baseline. This trial has been in compliance with the Helsinki Declaration and approved by the local ethics committee of Mid-Norway (Regional Ethics Committee number: 181–04) as well as the Norwegian Social Science Data Services, NSD (number: 11541).

### Instruments

This follow-up study followed a guided imagery intervention only 30 days after baseline [18,40] before a natural course the subsequent 14 years.

### Assessments

In 1991, the participants scored pain by a visual analogue scale (VAS) in two social contexts, i.e. initially in a doctor context (Pain DC) and subsequently in the home context (Pain HC) the same day. 14 years later 54.5% re-scored pain in the doctor context (Pain FU), see Figure 1.

A VAS scale for pain has shown to be reliable and valid [41], it ranges from 0–100 from zero to maximal pain, and it is recognized as sensitive to change [42]. At baseline participants also completed the Beck Depression Inventory (BDI), which is a 21-item self-report inventory designed to assess affective, behavioural, cognitive, motivational and vegetative aspects of depressive symptoms [43]. It has been shown to be a valid measure of depression, especially for the actual day of measurement [44]. Anxiety was measured by the State-Trait Anxiety Inventory-Trait (STAI-T), which consists of a set of 20 statements in which the respondent rates how anxious he/she feels on a four-point scale. Total scores range from 20 to 80 with high scores indicating a higher anxiety. The reliability of STAI-T is well documented [45]. The Millon Clinical Multiaxial Inventory (MCMI) was used as a psychological assessment tool to provide information on personality traits outlined in the DSM-system, e.g. cluster-c traits. It was developed and standardized specifically on clinical populations [46]. The variables considered in the study are specified in Table 1.

**Table 1:** Explanatory variables considered in the analysis

**Variables regarding**	**Variable type**	**Variable (numerical variable values, factor levels)**
Demographics	Numerical Factor	Age (yrs).
		Education (yrs).
		Single (no, yes).
Symptoms	Numerical	Insomnia (VAS)
		Pain duration (yrs).
		Anxiety (STAI-T)
		Depression (BDI)
	Factor	Use of amitriptyline (no, yes).
		Use of guided imagery (0 = none, 1 = visualization of landscape, 2 = visualization of pain control)
Personality, cluster- c	Numerical	Avoidant personality traits (MCMI-I)
		Dependent. personality traits (MCMI-I)
		OCD personality traits (MCMI-I)

### Data analysis

To examine differences between completers and non-completers, the significance was based on one-way ANOVA for the continuous variables and Pearson’s Chi-square and Fisher exact test for the categorical variables. To examine the inter-correlations between contextual and time-dependent pain, i.e. Pain HC (baseline), Pain DC (baseline) and Pain FU (Pain DC at follow-up), we used Pearson’s *r*. Dependent *t*-test were run to detect differences in the pain and mood variables (VAS 0–100) from Time and Context, while Fisher exact test tested the time effects for the categorical FM tender points data. In order to assess how the variables were associated with pain in the various contexts, linear models were fitted with pain (DC, HC and FU) as the response variables. Several explanatory variables obtained at baseline in the doctor context (DC) were considered (Table 1), and backwards stepwise regression was used to select models having a small number of variables.

In the regression model (which allowed categorical variables as well as numerical variables as explanatory variables), parameters were estimated by maximum likelihood using the glm function in the R system for statistical computation [47]. A backward-stepwise selection of terms based on F statistics was performed, aided by the drop term function of the MASS package for R [48]. The least significant term was omitted, and the procedure was repeated until all remaining terms contributed significantly to the model at the 0.05 level. Significance for the remaining baseline variables were based on standardized coefficients, each having a t distribution under the null hypothesis that the parameter is zero. Models were selected for doctor contextual pain (Pain DC), home contextual pain (Pain HC), and pain at follow-up (Pain FU). For the two latter, Pain DC was also included as an explanatory variable in the initial model.

## Results

None of the baseline variables showed any differences between the completers and the 14 years FU non-completers on one-way ANOVA for the continuous variables and Pearson’s Chi-square and Fisher exact test for the categorical variables. Pain improved with psychological treatment (guided imagery) after 30 days, as shown in the original study [18].

### Within-groups measures

#### Pain by context and time (long-term)

55 participants reported pain in both contexts at baseline. The mean contextual pain (VAS) difference for the completers (n = 30) at baseline was significant = 11.5 (t = 3.12, p = 0.004). For the whole sample (n = 55) at baseline, mean pain (VAS) was also lowest at home, i.e. a difference = 7.2 (t = 2.36, p = 0.022). Conversely, long-term time did *not* influence pain significantly after 14 years, showing a very small VAS difference from baseline to follow-up = 0.1 (p> 0.05) when measuring the completers in the same context (i.e. doctor context). See Table 2.

**Table 2:** **Pain reportings: Descriptives**^**a**^**and correlations**^**b**^**(n = 24)**

	**Mean**^**a**^**± SEM**	**(SD)**	**Baseline Doctor**	**Baseline Home**	**Follow-up 14 yrs Doctor**
Baseline Doctor	50.3^c d^ ± 4.0	21.3	-	0.49^b^ *	0.14 ^b^ ns
Baseline Home	38.8^c *^ ± 4.3	23.0	0.49 ^b^ *	-	0.47^b^ *
Follow-up 14 yrs Doctor	50.2^d^ ± 4.4	23.6	0.14^b^ ns	0.47^b^ *	-

^a^ Pain (VAS) descriptives.

^b^ Correlations (Pearson’s *r*), p values ≤ 0.05 are marked *

^c^ Dependent *t*-test of group means between baseline pain scores at home vs. in a doctor office; completers (* p<0.05).

^d^ Dependent *t*-test of group means between pains in doctor’s office with a 14 years’ time lag; completers (ns).

#### Mood by time

In contrast to pain, depression (BDI) and anxiety (STAI-T) both improved after 14 years from 16.7 to 12.5 (difference = 4.2) (t = − 2.52, df = 27, p < 0.05) and from 51.1 to 41.0 = 10.1 (t = −5.21, df = 27, p < 0.0001), respectively.

#### Tender points by time

Five out of 29 subjects (17.2%) had less than 11 of 18 tender points after 14 years, and thus *did not* fulfill to have FM anymore. Fisher's Exact Test for the count data indicated a significant change, p-value = 0.02; alternatively a true odds ratio is less than 1, with 95 percent confidence interval: 0.00-0.73.

### Correlations

The correlation between the two contextual dependent pain recordings Pain HC (baseline), Pain DC (baseline) was significant (*r* = 0.45), while the correlation between the two time dependent pain recordings Pain DC at baseline and Pain FU (Pain DC at follow-up) was not (r =0.14). See Table 2:

### Associations and predictor analyses

Baseline pain DC was positively associated with baseline trait anxiety (STAI-T) and insomnia scores. See Table 3. Baseline Pain HC was positively associated with baseline Pain DC, while amitriptyline appeared as a resilience factor. See Table 4.

**Table 3:** Model of predictors of pain at baseline (VAS) in doctor context from other baseline parameters, n = 26

**Parameter corresponding to**	**Estimate**	**Std. Error**	***t *value**	**Pr(> | *t *|)**
Intercept	- 9.23	19.81	- 0.47	0.65
Insomnia (VAS 0–100)	0.33	0.14	2.4	0.03
Anxiety (STAI-T)	.86	0.40	2.16	0.04

**Table 4:** Model of predictors of pain at baseline (VAS) in home context from other baseline parameters, n = 27

**Parameter corresponding to**	**Estimate**	**Std. Error**	***t *value**	**Pr (> | *t *|)**
Intercept	5.1143	9.0610	0.564	0.58
Pain in doctor context, Pain DC	0.7586	0.1645	4.611	0.00012
Amitriptyline	−18.952	7.8361	−2.419	0.02

Prediction of follow-up pain (Pain FU 14 years) from baseline variables showed that single-status, depression, long pain-duration and obsessive compulsive personality traits seemed to predict *worse* pain at follow-up 14 years ahead, while education and guided imagery coping strategies apparently predicted *less pain*, see Table 5:

**Table 5:** Model of predictors of pain at follow-up (VAS) after 14 years in a doctor context from the baseline parameters, N = 26

**Parameter corresponding to**	**Estimate**	**Std. Error**	***t *value**	**Pr(> | *t *|)**
Intercept	76.44	42.54	1.80	0.090
Education	- 8.25	2.53	−3.25	0.005
Single	35.73	12.67	2.82	0.012
Insomnia	- 0.55	0.22	- 2.51	0.023
Pain-duration	1.27	0.59	2.15	0.046
Depression (BDI)	1.17	0.48	2.45	0.026
Guided Imagery1	- 46.95	10.41	- 4.51	0.00031
Guided Imagery2	- 16.62	9.03	- 1.84	0.08 ns.
OCD personality traits	1.04	0.48	2.17	0.04

## Discussion

Mean pain was worse to a doctor than at home the same day, but appeared unchanged after 14 years in the same context. The pain recordings at the doctor’s office were not significantly correlated, indicating that pain was not stable within individuals, and our findings suggest that some factors may explain the individual differences for pain in context and time.

Pain measured to the doctor was positively associated with the *trait-anxiety* (STAI-T) and *insomnia* measures at baseline, findings which are in accordance with other studies about pain and anxiety [49]. For blood-pressure measurements, which tend to be higher to a doctor than at home, (the so-called white-coat syndrome), anxiety of previous visits to the physician’s office probably has an effect on subsequent recordings [26]. Previous research supports our findings that insomnia is associated with FM pain in the short-term perspective [3]. Until recently, it has been unknown whether poor sleep contributes to FM in the long-term. In a new study by Mork & Nilsen, sleeplessness predicted fibromyalgia during 10 years in a previous healthy population [50].

When pain was measured *at home* the same day, previous pain mattered for pain worsening, a finding in accordance with other studies [13,51], while amitriptyline appeared as a resilience factor. Some analogue studies have shown that drug-effects may depend on their context, the so called place preference or place aversion (classical) conditioning [33,52].

Baseline depression, long pain-duration, being single and OCD-traits were associated with worse pain 14 years ahead. These findings support previous literature that depression is a risk factor for long-term FM symptoms, e.g. [14]. However, depression may act both as a risk factor and a consequence of pain [53] and vary with third factors [54,55]. Self-critical perfectionism, an active generation of stress, stress sensitivity and levels of depression are features which may explain the association between OCD and forthcoming pain [56]. In contrast, education seemed to be a resilience factor for future pain in our study, a finding in accordance with previous findings that FM patients have lower educational level than non-patients, e.g. [57]. Guided imagery also appeared as a resilience factor in this 14 years follow-up of the Fors et al. 2002 study [18], which supports findings that relaxation, imagery and cognitive training may predict reductions in pain compared to untreated patients [58]. Syrjala et al. [59] and van Kuiken et al. [60] have revealed results which show that guided imagery have positive effects the first five to seven weeks after treatment, but that the effects seem to yield after 18 weeks [61] in reducing persistent pain [19,62], but it has not been investigated in a long-term prospective studies previously.

This study brings new information about how pain vary with context among patients with FM. A strength of the study was that pain was measured in two different contexts the same day and two similar contexts with long term follow up.

Several limitations of this study needs to be addressed: The statistical model used for associations and prediction of pain (in a doctor context, in a home context, and in a doctor context after 14 years) was an ANCOVA linear regression model, which was fitted to the data using a backwards stepwise selection using F-tests with 0.05 as critical value, so that we ended up with a model where each covariate was significant in presence of the others. The low number of participants (n = 29), leading to low power may have prevented discovery of potential predictors. Also, the strict significance level of 0.05 may have possibly omitted important predictors from the models. On the other hand, the multiple testing procedure to select covariates in stepwise regression in general may lead to too small p-values and possibly false findings. Hence the findings may be regarded as hypothesis-generating findings more than true predictions. There was great individual variability among the pain sufferers, which may explain why individual baseline current pain did not predict future pain. Another weakness in the study was that the frequency of the guided imagery use was not measured, so the effect of this intervention may be spurious. Another possible limitation of our study was the measurement of just current pain. In chronic pain studies many investigators measure pain scores as the mean sum of current+ last week + maximal + minimal pain, as seen in e.g. the Brief Pain Inventory [63]. However, we would expect a current pain measure to be more sensitive to variations in context than an aggregated measure of pain. Thus, contextual pain cues should be accounted for when pain is monitored and treated, e.g. by focusing more on home-measured pain and doctor office context.

## Conclusion

Our study suggests that contexts should be accounted for when reporting current pain. It seems to matter where and how the pain is recorded. Our study also suggests that the factors explaining the variance in pain may vary by contexts. The implication of our findings would advocate a new approach to pain by focusing more on home-measured pain and modifying the context or environment of the doctors’ office if possible, e.g. to emphasize comfort and empathy more [64] in order to reduce pain.

## Abbreviations

FM: Fibromyalgia; VAS: Visual analogue scale; ACR: American College of Rheumatology; DC: Doctor context; HC: Home context; FU: Follow-up; CWP: Chronic widespread pain; TP: Tender points; CBT: Cognitive behavioral therapy; QoL: Quality of life; PET: Positron emission tomography; fMRI: Functional magnetic resonance imaging or functional MRI.

## Competing interests

The authors declare that they have no competing interests.

## Authors’ contributions

EAF conceived and designed the study and drafted the first version of the manuscript. TL and ØB participated in the design of the study and TL participated in the collection of data and coordination of the study ØB was responsible for the statistical analyses, and EAF and TL contributed in analyzing the data. TL and ØB contributed in writing the paper. All authors read and approved the final manuscript.
